# Stimulus Threat and Exposure Context Modulate the Effect of Mere Exposure on Approach Behaviors

**DOI:** 10.3389/fpsyg.2016.01881

**Published:** 2016-11-29

**Authors:** Steven G. Young, Isaiah F. Jones, Heather M. Claypool

**Affiliations:** ^1^Baruch College, City University of New York, New YorkNY, USA; ^2^Electronic Arts, Global Analytics and Insights, Redwood CityCA, USA; ^3^Department of Psychology, Miami University, OxfordOH, USA

**Keywords:** mere exposure effect, approach avoidance, familiarity, emotions, expression

## Abstract

Mere-exposure (ME) research has found that initially neutral objects made familiar are preferred relative to novel objects. Recent work extends these preference judgments into the behavioral domain by illustrating that mere exposure prompts approach-oriented behavior toward familiar stimuli. However, no investigations have examined the effect of mere exposure on approach-oriented behavior toward threatening stimuli. The current work examines this issue and also explores how exposure context interacts with stimulus threat to influence behavioral tendencies. In two experiments participants were presented with both mere-exposed and novel stimuli and approach speed was assessed. In the first experiment, when stimulus threat was presented in a homogeneous format (i.e., participants viewed exclusively neutral or threatening stimuli), ME potentiated approach behaviors for both neutral and threatening stimuli. However, in the second experiment, in which stimulus threat was presented in a heterogeneous fashion (i.e., participants viewed both neutral and threatening stimuli), mere exposure facilitated approach only for initially neutral stimuli. These results suggest that ME effects on approach behaviors are highly context sensitive and depend on both stimulus valence and exposure context. Further implications of these findings for the ME literature are discussed.

## Introduction

Past research has documented that a range of positive affective and evaluative reactions are induced via unreinforced exposure [i.e., mere-exposure (ME), Zajonc, 1968]. This ME effect is robust and occurs for stimuli including asocial shapes (e.g., Kunst-Wilson and Zajonc, 1980), faces (e.g., Claypool et al., 2007), and experimental confederates (Bornstein et al., 1987), and when stimulus exposure is readily perceptible or subliminal (see Bornstein, 1989, for a review). Moreover, under some circumstances, ME improves attitudes toward stimuli initially perceived as potentially threatening (e.g., Litvak, 1969; Young and Claypool, 2010).

This preference for familiarity has been explained through various means. For example, some theories have suggested a preference for familiar objects is the result of repeated exposure reducing the perceived threat associated with novel stimuli (e.g., Bornstein, 1989). Indeed, there is evidence that ME reduces arousal (i.e., anxiety) as measured via skin conductance (Zajonc, 1968), that familiar stimuli are especially enjoyed under conditions of uncertainty (Lee, 2001), and that ME effects are especially strong for those relativity high in trait anxiety (Harmon-Jones and Allen, 2001; but see Ladd and Gabrieli, 2015). A separate avenue of research has examined how differences in the subjective experience of processing novel and familiar stimuli contribute to ME effects. Specifically, this line of work finds that repeated exposure allows for fluent processing (e.g., Bornstein and D’Agostino, 1994), and this fluency is affectively positive (e.g., Reber et al., 2004), as seen on self-report and physiological outcome measures (e.g., facial muscle movements indicating smiling, Winkielman and Cacioppo, 2001). Though the ME effect is well-documented and likely reflects co-acting mechanisms (e.g., preference for “safe” familiar objects and fluent processing), other theoretically important questions remain under-explored. One such question concerns the impact of ME on approach behavior. If ME improves attitudes toward associated stimuli, then it might facilitate one’s desire to physically approach said objects. Consistent with this reasoning, preliminary research shows that ME encourages one to approach familiar (relative to novel) stimuli (Jones et al., 2011), offering initial evidence that ME relates to basic approach motivations. Yet, much more needs to be uncovered about the robustness and possible limiting factors of these effects. For example, does ME to initially threatening stimuli also facilitate approach responses? And, if so, does this occur under only limited circumstances? The current work explores both of these questions.

### Mere Exposure, Approach, and Stimulus Threat

Given the positivity engendered by ME, a logical prediction is that familiarity should encourage approach-related behaviors. Jones et al. (2011) provided evidence in support of this hypothesis. Participants were first shown a series of initially neutral objects (e.g., cups, tools) and later these same (now familiar) objects again intermingled with a set of novel neutral objects. On each trial, participants were directed to either approach or avoid the object on the screen by utilizing a joystick (e.g., Chen and Bargh, 1999). The findings indicated that participants were faster to make approach motions in response to familiar objects relative to novel ones. In a follow-up study, instead of being directed to approach or avoid familiar and novel objects, participants freely choose which behavior to enact. Findings showed that familiar objects were approached more frequently than novel ones.

Studies such as these extend the scope of ME effects to include behavioral measures of approach and avoidance. However, these experiments focused exclusively on neutral stimuli, leaving unanswered whether these effects generalize to other objects. Yet, there is reason to suspect that behavioral outcomes of ME are possible even for stimuli initially perceived as threatening. The reason for this assertion is straightforward: although relatively few studies have explored ME effects on threatening stimuli, there is evidence to suggest that reactions to negative stimuli can be altered by familiarity, implying that approach behaviors may be malleable too.

For example, Litvak (1969) twice exposed participants to a live snake and found that initial attitudes toward the snake improved after ME. Moreover, Bornstein (1989) suggested that ME may aid in the treatment of phobias, and many phobias do in fact respond favorably to exposure therapies (e.g., Emmelkamp, 2003). In social contexts, Bornstein’s (1993) meta-analytic investigation illustrated that ME to racial outgroups (stimuli that are often initially perceived as threatening) reduced prejudice. More recently, Young and Claypool (2010) found that ME to angry (threatening) faces led participants to rate familiar angry faces as less negative than novel angry faces and also reduced attention allocation to familiar, relative to novel, angry faces, suggesting that fear responses indicated by attentional engagement (Öhman et al., 2001) are attenuated by virtue of ME (see also, Stone and Valentine, 2005).

Such research indicates that ME can influence affective, evaluative, and attentional processes by reducing negativity toward initially aversive stimuli. Yet, the implications of these evaluative and attentional effects for approach behaviors are untested. Given the evidence that ME facilitates approach behavior toward previously exposed neutral stimuli (e.g., Jones et al., 2011), and that ME can overcome negative reactions to initially threatening stimuli (e.g., Young and Claypool, 2010), one possibility is that ME to threatening objects will similarly facilitate approach toward them.

Alternatively, the possibility exists that ME alone is insufficient to overcome avoidant cues afforded by threatening stimuli. Indeed, a powerful link exists between stimulus threat and avoidance reactions (e.g., Duckworth et al., 2002; Marsh et al., 2005) suggesting that, though ME might modulate affective reactions toward such stimuli, these effects might not extend to actual approach/avoidance behavior. Furthermore, given that repeated exposure can lead to fluent processing (e.g., Bornstein and D’Agostino, 1994), this fluency may serve to make the dangerous implications of threatening stimuli clearer, potentially overwhelming the normally positive effects of ME (Reber et al., 2004). Thus, one aim of the current research is to test these competing predictions.

Given these competing hypotheses about the impact of familiarity on approach behaviors toward threatening stimuli, it is possible that situational and contextual moderators may dictate when one outcome is possible versus the other. Indeed, the link between familiarity and liking is not immutable. For example, perceiver states, such as positive and negative moods, can modulate the preference for familiar stimuli (de Vries et al., 2010), as can stimulus features like category prototypicality (e.g., Halberstadt, 2006).

One contextual element of particular relevance to the current work concerns the heterogeneous versus homogenous presentation of stimuli. The value of familiarity is obfuscated when repeated stimuli are not interspersed with novel stimuli (Bornstein, 1989). For example, Dechene et al. (2009) first exposed participants to a set of symbols in an exposure task. Later, some participants saw these same (repeated) symbols intermixed with a set of novel symbols. Other participants, however, saw a list of “all-old” (repeated) symbols or saw a list of “all-new” (non-repeated) symbols. In the intermixed condition, participants reported liking repeated symbols more than novel ones (i.e., a ME effect). However, participants’ liking ratings in the “all-old” condition were *not* different than those in the “all-new” condition. The explanation for these findings is that viewing both old and new stimuli enhances the signal strength of familiarity, making the perceptual and affective differences between novel and repeated stimuli more salient. In a mixed-presentation context, the familiar stimuli *feel different* from the new stimuli, causing robust ME effects. In homogenous presentation contexts, this subjective experience is dulled and thus familiarity has little impact on affective reactions.

Based on this work, it is clear that the impact of ME itself varies based on presentation context. Extending this logic, we speculate that ME’s impact on reactions to initially threatening stimuli may vary by presentation context as well. Here, though, we are concerned with the homogenous (versus heterogeneous) presentation *of threat itself* (cf. Dechene et al., 2009). Our hypothesis is that, when the initial stimulus-threat is homogenous (i.e., participants encounter exclusively threatening or non-threatening stimuli), the relative fluency/familiarity of the stimuli will be more salient than the threat itself, as familiarity will be the only source of variability in the stimuli. In such cases, we predict that familiarity with both initially threatening and novel stimuli will facilitate approach behaviors. Such results would replicate Jones et al. (2011) findings with neutral stimuli while demonstrating that ME can facilitate approach to negative stimuli as well (a noteworthy and novel extension). We believe that, if one *must* deal with potential threats, it may be preferable to deal with a familiar threat, which may be judged as slightly more favorable (or slightly less distressing) because it has proven harmless on prior encounters (e.g., Young and Claypool, 2010).

Our hypothesis when participants encounter *both* threatening and non-threatening stimuli is different. For stimuli that are initially non-threatening, we predict that ME will facilitate approach responses. However, for initially threatening stimuli, we predict that ME will no longer do so. When threatening stimuli are intermixed with neutral stimuli, stimulus threat *and* familiarity vary across stimuli, allowing participants to attend to both. In mixed contexts, perceivers are not forced to deal *only* with threats and therefore may be less sensitive to the familiarity or novelty of negative stimuli, but more attuned to their negativity. This will likely cause the signal strength of ME to be muted, dampening any approach tendencies.

### The Current Research

We hypothesized that (1) ME to initially threatening stimuli will potentiate approach behaviors when stimulus threat is presented in a homogenous context, but that (2) such effects will be eliminated when it is presented in a mixed context. We also hypothesized that (3) ME to initially neutral stimuli will potentiate approach in both homogenous- and mixed-threat contexts. Hypotheses 1 and 3 were tested in the first experiment, in which stimulus threat was manipulated between-subjects, such that participants viewed familiar and novel stimuli that were either exclusively non-threatening (neutral-expression faces) or, instead, exclusively threatening (angry faces). Hypotheses 2 and 3 were examined in a follow-up experiment in which stimulus threat was manipulated within-subjects, creating a situation wherein stimulus threat varied within perceivers.

## Experiment 1

### Participants

Thirty-eight introductory psychology students participated in exchange for partial course credit. Sample size was based on observed effect sizes in similar work (Jones et al., 2011). The research was approved by the Miami University ethics committee, and all participants were recruited from the participant pool.

### Materials

Approach and avoidance tendencies were measured with a joystick (Logitech Extreme 3-D Pro). Stimulus threat was manipulated on a between-subjects basis using 16 photographs of faces from the NimStim database (Tottenham et al., 2009). The same faces, displaying each of the two emotions (neutrality and anger), were used across conditions.

### Procedure

The procedure of Experiment 1 closely followed Jones et al. (2011, Experiment 1). After providing consent, participants were seated in front of a computer in individual cubicles and informed that they were taking part in a study investigating motor responses. During the initial exposure phase, participants viewed eight stimulus faces one time, each for 1 s, in a random order. Depending on condition, all photos depicted either neutral or angry (threatening) faces. All faces were sized to 250 × 250 pixels. After this phase, participants engaged in a roughly 5-min filler task in which they named the capital of each US state.

Next, participants began the second phase of the study in which approach and avoidant behaviors were assessed. During this phase, participants viewed the eight (familiar) faces from the first phase randomly intermixed with eight novel faces. Each trial began with presentation of the stimulus photo for 1 s. Then, the word “PUSH” or ”PULL” randomly appeared in the center of the screen until the participant, using his/her dominant hand, performed the corresponding motion with the joystick. The time participants needed to move the joystick to its apex was recorded. Trials where participants failed to fully push/pull the joystick to the end of its range were recorded as errors. To firmly establish an “object frame of reference” (in which pushes [pulls] of the joystick equate to approach [avoidance] behavior, e.g., Seibt et al., 2008), participants were instructed that they would be moving the joystick toward or away from the *computer screen*. One second after the participant completed the instructed behavior, the next trial began. After completing this task, participants were debriefed and dismissed^1^.

Participants completed 16 trials; on eight trials they pushed the joystick toward the screen (approached the stimulus), four each in response to familiar and novel stimuli. Similarly, they pulled the joystick away from the screen (avoided the stimulus) on eight trials, four each in response to familiar and novel stimuli. Thus, the experiment employed a 2 (expression: neutral, angry) × 2 (motion: push, pull) × 2 (status: familiar, novel) mixed design, with the latter two factors manipulated within-subjects. Between subjects, we counterbalanced which set of stimuli was approached/avoided and which was familiar/novel. These counterbalancing factors yielded no significant main effects or interactions, and they were dropped from all subsequent analyses.

### Results and Discussion

#### Data Transformations

Five individual trial responses (<1% total) were removed because of extreme reaction time latencies (i.e., below 300 ms [0 responses] and above 3000 ms [5 responses]). Additionally, one participant was removed as an outlier because his or her mean reaction time in one of the cells of the design was greater than ±3 SDs from the mean, leaving a final sample of 37.

#### Analyses

**FIGURE 1:**
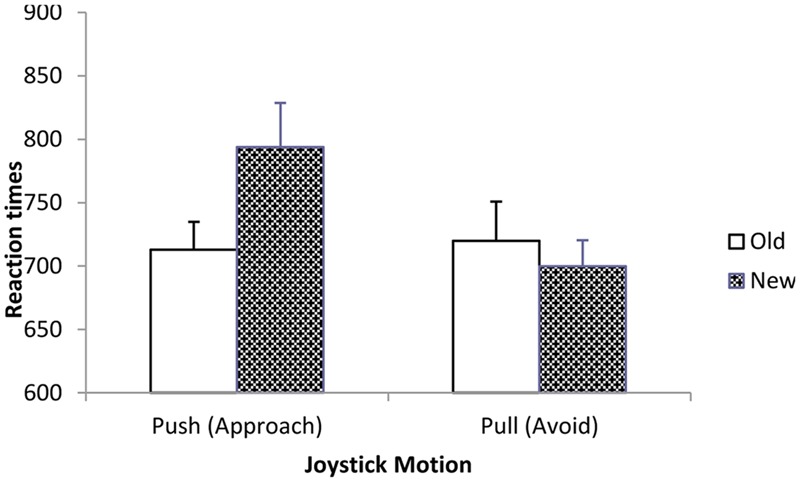
**Reaction times to perform pushing and pulling motions in response to familiar (Old) and novel (New) targets (Experiment 1)**.

Reaction times to make the instructed joystick motion were subjected to a 2 (expression) × 2 (motion) × 2 (status) mixed-model ANOVA. This analysis revealed a main effect of motion; participants pulled the joystick (*M* = 708, *SD* = 147) faster than they pushed (*M* = 753, *SD* = 151), *F*(1,34) = 5.06, *p* = 0.03, *d* = 0.34. More theoretically important, a motion × status interaction emerged, *F*(1,34) = 6.95, *p =* 0.01, *d* = 0.43 (see **Figure 1**). To examine our hypotheses, we conducted simple effects tests to determine the impact of familiarity for each type of behavioral response. Simple effects showed that when participants were directed to push forward (approach), they did so more quickly after viewing familiar (*M* = 713, *SD* = 133) than novel (*M* = 793, *SD* = 211) faces, *F*(1,34) = 6.78, *p* = 0.01, *d =* 0.43. Also, when directed to pull away from (avoid) the face, they did so more quickly in response to novel (*M* = 701, *SD* = 124) than to familiar (*M* = 718, *SD* = 188) faces, though this effect did not approach significance, *F*(1,34) = 0.67, *p* = 0.42.

**FIGURE 2:**
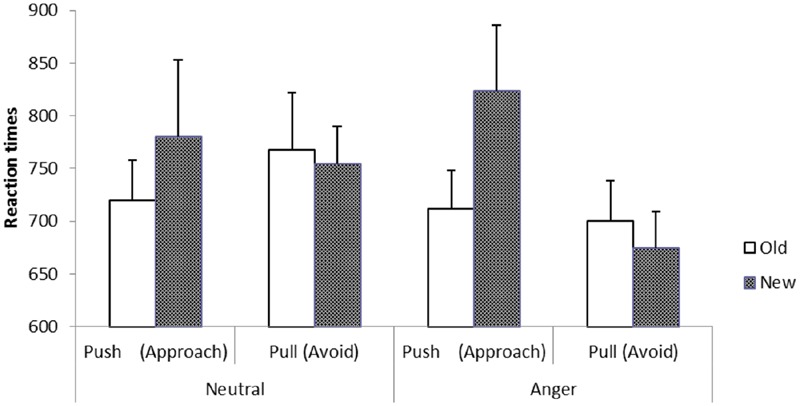
**Reaction times to perform pushing and pulling motions in response to familiar (Old) and novel (New) targets for each stimulus type (Experiment 1)**.

Finally, as predicted, stimulus threat (expression) did not moderate the behavioral patterns described above, as the three-way interaction did not approach significance, *F*(2,34) = 0.31, *p* = 0.74. As shown in **Figure 2**, mere exposure potentiated approach when stimuli were initially neutral and when stimuli were initially threatening.

Consistent with Jones et al. (2011), Experiment 1 found that participants were quicker to approach familiar than novel stimuli. Notably, these behavioral tendencies appear to generalize to both neutrally expressive and angry (threatening) faces. The current results supported hypothesis 1.

## Experiment 2

In Experiment 2, we test the hypothesis that the ability of ME to potentiate approach responses for threatening stimuli is limited to homogenous threat contexts. Specifically, we hypothesized that, when perceivers face both threatening and neutral objects, ME to threatening stimuli will no longer potentiate approach. To test this, stimulus threat was manipulated within-subjects to create a situation in which perceivers were faced with both threatening and non-threatening stimuli that varied in their familiarity. We predicted that ME would continue to potentiate approach responses after participants viewed non-threatening stimuli, but would have no impact on approach speed following the presentation of initially threatening stimuli.

### Participants

Forty-one introductory psychology students participated in exchange for partial course credit. The research was approved by the Miami University ethics committee, and all participants were recruited from the participant pool.

### Materials and Procedure

The faces displaying angry and neutral expressions used in Experiment 1 were again used in Experiment 2. The procedures were identical to Experiment 1, except where noted. First, face expression was manipulated within-subjects. Second, behavioral responses were measured during both the initial exposure phase (block 1) and during a second block. Thus participants responded twice to all stimuli, once when they were novel and once again when they were familiar. This adjustment to the joystick procedure was introduced for several reasons. First, it allowed us to record more reaction times for each participant, potentially providing more stable means and reliable measures of approach and avoidance tendencies. Second, by recording behavioral responses after the initial and repeated viewing of each stimulus, we can examine directly how behavioral responses to neutral and negative stimuli change from initial exposure to repeated exposure. This provides a dynamic and sensitive measure of approach/avoidance that cleanly tests the impact of familiarity on behavioral tendencies.

During Block 1, participants viewed 16 faces (8 neutral, 8 angry), one at a time, each for 1 s, presented in a random order. Immediately after face presentation, participants were instructed to either “Push” or “Pull” the joystick, and these instructions remained on screen until participants responded. Participants “pushed” after the presentation of four angry and neutral faces, and “pulled” after the presentation of four angry and neutral faces. Participants next completed the same filler task as in Experiment 1 before completing the second behavioral block, which was identical to the first block, and all faces were followed by the same behavioral instructions [i.e., if a face was paired with “push” (approach) in the first block, it was again paired with “push” (approach) in the second]. Which faces were angry/neutral and which were approached/avoided was counterbalanced between subjects. No counterbalancing factors interacted with the theoretically meaningful effects reported below, thus they were removed from all further analyses. The design of the experiment was a 2 (expression: neutral, angry) × 2 (motion: push, pull) × 2 (status: familiar, novel) within-subjects design.

### Results and Discussion

#### Data Transformations

Individual trial responses were dropped using the same criteria as Experiment 1, resulting in 19 individual responses being dropped. One participant was removed as an outlier based on reaction time latencies. Additionally, data from two participants were lost due to computer error, leaving a final sample of 38.

#### Analyses

**FIGURE 3:**
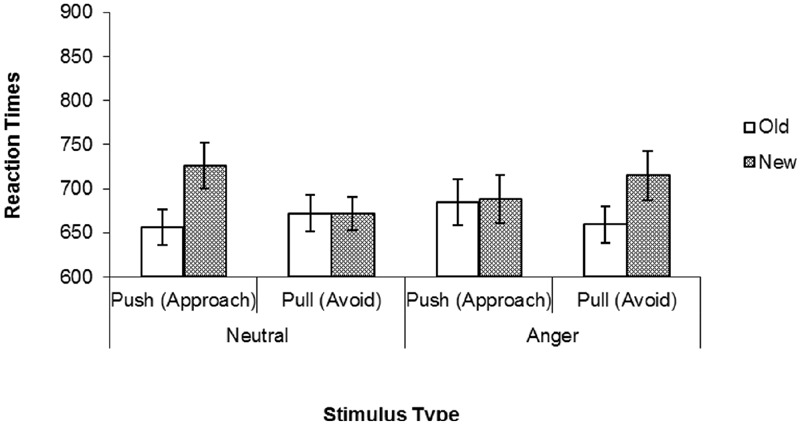
**Reaction times to perform pushing and pulling motions in response to familiar (Old) and novel (New) targets for each stimulus type in Experiment 2**.

Reaction times to make the instructed joystick motion were subjected to a 2 (expression) × 2 (motion) × 2 (status) repeated-measures ANOVA. This analysis revealed a main effect of status; participants responded faster overall to the familiar (*M* = 664, *SD* = 93) compared to the novel faces (*M* = 695, *SD* = 195), *F*(1,37) = 5.65, *p* = 0.023. More theoretically important, the three-way interaction emerged, *F*(1,37) = 4.25, *p =* 0.05, *d =* 0.33 (see **Figure 3**). To explore this three-way interaction, separate 2 (motion) × 2 (status) ANOVAs were conducted for angry and neutral face stimuli.

For neutral stimuli, there was the same main effect of status discussed above, *F*(1,37) = 7.52, *p* = 0.009, which was qualified by a significant status × motion interaction, *F*(1,37) = 4.21, *p* = 0.05 *d =* 0.33. Consistent with Experiment 1, simple effects found that participants were faster to make approach motions following familiar neutral (*M* = 653, *SD* = 108) than novel neutral (*M* = 720, *SD* = 144) faces *F*(1,37) = 10.27, *p* = 0.003. *d* = 0.51. However, there was no difference in avoidance time in response to familiar (*M* = 663, *SD* = 109) and novel (*M* = 670, *SD* = 101) neutral stimuli, *F*(1,37) = 0.11, *p* = 0.74.

Alternatively, for angry faces, the status × motion interaction was not significant *F*(1,37) = 1.31, *p* = 0.26, and there were no significant main effects of motion (*p* > 0.84) or status (*p* > 0.15). For angry (threatening) faces, ME did not potentiate subsequent approach behavior. Regarding neutral stimuli, Experiment 2 replicates our previous results, as familiar faces potentiated approach behaviors, relative to novel faces. Unlike Experiment 1, however, these effects were not found for threatening faces. Such results indicate that when participants view homogeneous stimulus sets (e.g., all angry or all neutral faces, as in Experiment 1), this may render stimulus threat less salient than the relative familiarity of the previously seen stimuli. However, when stimulus threat is presented heterogeneously, this likely amplifies the differential affective features of the stimuli and draws a sharper distinction between the behavioral reactions prompted by either neutral or dangerous objects. Experiment 2 therefore highlights an important boundary condition for the influence of familiarity on approach and avoidance behaviors.

## General Discussion

Evaluations of initially neutral stimuli have been consistently shown to improve as a result of mere exposure (e.g., Bornstein, 1989) leading to approach behaviors toward familiarized neutral stimuli (Jones et al., 2011). Evidence also suggests that negative stimuli are found less threatening following ME (Litvak, 1969; Young and Claypool, 2010). However, the extent to which these effects on threatening stimuli extend to behavioral outcomes is untested. Moreover, the familiarity-positivity link is subject to numerous contextual factors, including the exposure context (Dechene et al., 2009). Thus, the current research aimed to test whether ME to threatening stimuli can influence approach behaviors and whether such behavioral outcomes are modulated by the exposure context.

In Experiment 1, stimulus threat had no impact on the influence of familiarity on approach and avoidance behaviors when participants were exposed to exclusively threatening stimuli. However, when participants were exposed to *both* threatening and neutral objects together (Experiment 2), the behavioral outcomes found in the first experiment and in Jones et al. (2011) replicated for neutral stimuli, yet failed to materialize for threatening stimuli. Thus, under conditions where stimulus valance is held constant and only familiarity (vs. novelty) differs, the signal strength of familiarity appears to trump the valence of the stimuli. However, viewing threatening stimuli together with neutral objects resulted in ME facilitating approach behaviors following the presentation of initially neutral stimuli only, suggesting that increasing the salience of an object’s threat by presenting it in concert with neutral stimuli mitigates the ability of a single previous exposure to influence behavior. This makes sense, insofar as ME signals safety and reduces negative reactions toward familiarized threats (e.g., Litvak, 1969; Young and Claypool, 2010) then individuals dealing with uniformly negative stimuli may be more attuned to information conveying the relative threat of one stimulus over another. However, when participants are exposed to stimuli that vary in both initial valence and familiarity, the differential affective qualities and behavioral affordances of the stimuli may become more salient, facilitating approach behaviors only after the presentation of familiarized neutral stimuli.

An additional consideration is what, if anything, the current the results suggest about underlying mechanisms of the ME effect. As noted earlier, some theories have suggested that novelty cues an immediate wariness that is tempered by familiarity (e.g., Zajonc, 1968; Young and Claypool, 2010), while other theories have instead emphasized the increased processing fluency of familiarized stimuli (e.g., Bornstein and D’Agostino, 1994; Reber et al., 2004). In our view, these are not incompatible explanations of the ME effect. To the extent that fluency is a heuristic cue to familiarity (e.g., Whittlesea, 1993), fluency may be positively marked because it suggests a stimulus has been previously encountered without harm. As such, our findings follow from both explanations of ME effects. Moreover, our work was not designed as a test between the two competing accounts ME effects.

However, we did test competing predictions derived from the literature regarding whether familiarity with threatening stimuli would reduce their negativity, likely by signaling safety (e.g., Young and Claypool, 2010) versus amplify their negativity because fluency allows for more ready access to the threatening information (Reber et al., 2004). The results are largely consistent with prior work showing that threat is reduced by familiarity (e.g., Litvak, 1969; Harmon-Jones and Allen, 2001; Young and Claypool, 2010), rather than amplified. The critical qualification observed presently is that exposure context determines whether familiar tempers negativity – operationalized as approach/avoidance tendencies currently – or whether stimuli valence alone directs behavioral responses.

### Limitations and Future Directions

Though the current work provides evidence that ME readies specific behavioral responses following exposure to neutral and threatening stimuli under certain circumstances, our results are not without limitations. First, the current experiments did not include a classic evaluative measure of the ME effect. That said, similar research (e.g., Young and Claypool, 2010; Jones et al., 2011) has found changes in evaluations of both neutral and threatening stimuli following ME. Combined with the large literature documenting evaluative shifts resulting from ME (Bornstein, 1989), we are confident that this current design adequately manipulated affective reactions toward the stimuli.

There is also a consistent pattern of data across both experiments that deserves mention: novel negative stimuli were never avoided faster than novel neutral stimuli. While this appears inconsistent with research exploring behavioral outcomes of stimulus evaluation, an important point to note is that much of this past work has revealed approach and avoidance differences between stimuli that were negative and positive (e.g., Chen and Bargh, 1999; Rotteveel and Phaf, 2004). However, the diminished difference in behavioral affordances and affective reactions between negative and neutral stimuli in the current work may explain why novel negative stimuli were not avoided faster than novel neutral stimuli.

A related issue regarding the current findings is that in both experiments participants were never slower to avoid familiar objects than novel ones. That is, though ME facilitated approach, it did not slow avoidance. Interestingly, this same pattern was also evident in Jones et al. (2011), suggesting that ME’s impact on behavior may be approach-specific. This speculation is bolstered by work showing that both positive and negative affective reactions as well as approach and avoidance reactions are mediated by different neural substrates (e.g., Davidson, 1992; Maxwell and Davidson, 2007). Thus, any factor (like ME) could have an impact on approach without necessarily having an impact on avoidance.

## Conclusion

The current work offers several interesting contributions. For instance, the finding that ME modulates basic approach/avoidance responses (Jones et al., 2011) is extended in two important ways. First, this effect is extended to threatening stimuli, which have only occasionally been included in ME research (e.g., Litvak, 1969) and never in a behavioral study. Second, the current results find that behavioral effects of ME on threatening (but not neutral) stimuli are sensitive to the context in which participants are exposed to the stimuli. Collectively, these results extend the existing ME literature and provide additional insight into how the context sensitivity of ME generalizes across stimulus valence and influences behavior.

## Author Contributions

All authors contributed to experiment design and manuscript preparation. SY analyzed the data.

## Conflict of Interest Statement

The authors declare that the research was conducted in the absence of any commercial or financial relationships that could be construed as a potential conflict of interest.

## Footnotes

1 The current experimental set up is identical to that employed in our previous work (Jones et al., 2011), using the same joysticks, monitors, and cubicles for data collection. In this previous paper, data were reported confirming that participants construed pushing motions as “approach” and pulling motions as “avoidance,” indicating an object-reference orientation (e.g., Seibt et al., 2008). We refer interested readers to Jones et al. (2011) for full details.
